# Indigenous species barcode database improves the identification of zooplankton

**DOI:** 10.1371/journal.pone.0185697

**Published:** 2017-10-04

**Authors:** Jianghua Yang, Xiaowei Zhang, Wanwan Zhang, Jingying Sun, Yuwei Xie, Yimin Zhang, G. Allen Burton, Hongxia Yu

**Affiliations:** 1 State Key Laboratory of Pollution Control & Resource Reuse, School of the Environment, Nanjing University, Nanjing, P. R. China; 2 Nanjing Institute of Environmental Sciences, Ministry of Environmental Protection, Nanjing, China; 3 School for Environment and Sustainability, University of Michigan, Ann Arbor, MI, United States of America; National Taiwan Ocean University, TAIWAN

## Abstract

Incompleteness and inaccuracy of DNA barcode databases is considered an important hindrance to the use of metabarcoding in biodiversity analysis of zooplankton at the species-level. Species barcoding by Sanger sequencing is inefficient for organisms with small body sizes, such as zooplankton. Here mitochondrial *cytochrome c oxidase I* (*COI*) fragment barcodes from 910 freshwater zooplankton specimens (87 morphospecies) were recovered by a high-throughput sequencing platform, Ion Torrent PGM. Intraspecific divergence of most zooplanktons was < 5%, except *Branchionus leydign* (Rotifer, 14.3%), *Trichocerca elongate* (Rotifer, 11.5%), *Lecane bulla* (Rotifer, 15.9%), *Synchaeta oblonga* (Rotifer, 5.95%) and *Schmackeria forbesi* (Copepod, 6.5%). Metabarcoding data of 28 environmental samples from Lake Tai were annotated by both an indigenous database and NCBI Genbank database. The indigenous database improved the taxonomic assignment of metabarcoding of zooplankton. Most zooplankton (81%) with barcode sequences in the indigenous database were identified by metabarcoding monitoring. Furthermore, the frequency and distribution of zooplankton were also consistent between metabarcoding and morphology identification. Overall, the indigenous database improved the taxonomic assignment of zooplankton.

## Introduction

Planktonic organisms play vital roles in food webs, biogeochemical cycles and other aquatic ecosystem functions [1]. Furthermore, due to their rapid responses to environmental variation, planktonic organisms have been used as indicators of ecosystem changes [2]. Despite its ecological importance, our understanding of the biodiversity of these organisms is hindered by difficulties in their identification which is complicated, time-consuming and requires unique expertise [3, 4].

The advent of high-throughput sequencing has provided an alternative to overcome issues associated with morphology-based biomonitoring. In recent years, high-throughput sequencing has resulted in dramatic advances in practical, cost-effective molecular approaches to analysis of environmental samples. Metabarcoding has several applications [5], such as investigating biodiversity [6], characterizing prey diversity in gut contents [7], and analyzing food-web dynamics [8]. Zooplankton are well suitable for metabarcoding analysis, because of their wide distribution in water and easiness of sampling. Recent applications of metabarcoding provided useful information on the genetic diversity of freshwater and marine planktonic organism communities [9, 10]. Nevertheless, functional assessment of communities and biodiversity by metabarcoding is constrained because of the limited reference barcode databases [11]. In some studies, more than 40% of the obtained operational taxonomic units (OTUs) could not be confidently assigned to a taxonomic group [7, 12].

Another problem is that the DNA crude extract obtained from a digested zooplankton [13] is contaminated by gut prey and intracellular endosymbiotic bacteria (e.g., *Wolbachia*) [14, 15]. The single sequence from Sanger sequencing can be the product of co-amplification of contaminated DNA and may not represent the ‘true’ barcode of the target individual. This DNA contamination leads to a noisy signal and confuses the barcode sequence capture [5]. High-throughput sequencing allows for sequencing millions of DNA fragments in parallel, significantly increasing sample throughput and process efficiency. Additionally, high-throughput sequencing allows for generation of multiple sequences for a single sample and provides an opportunity to identify the contamination of prey and endosymbiotic bacteria [16]. The use of high-throughput sequencing, therefore, overcomes some of the inherent limitations of Sanger sequencing for barcoding small body size organism [5].

**Fig 1:**
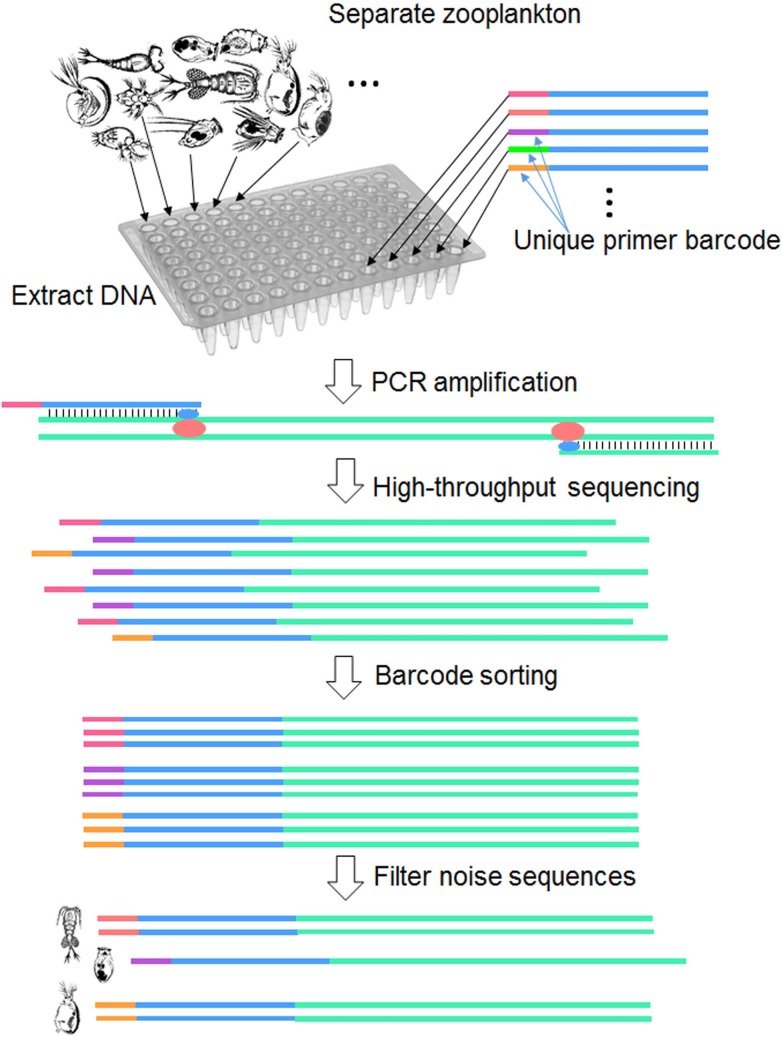
Schematic diagram of parallel barcode recovery using a high throughput sequencing protocol.

**Fig 2:**
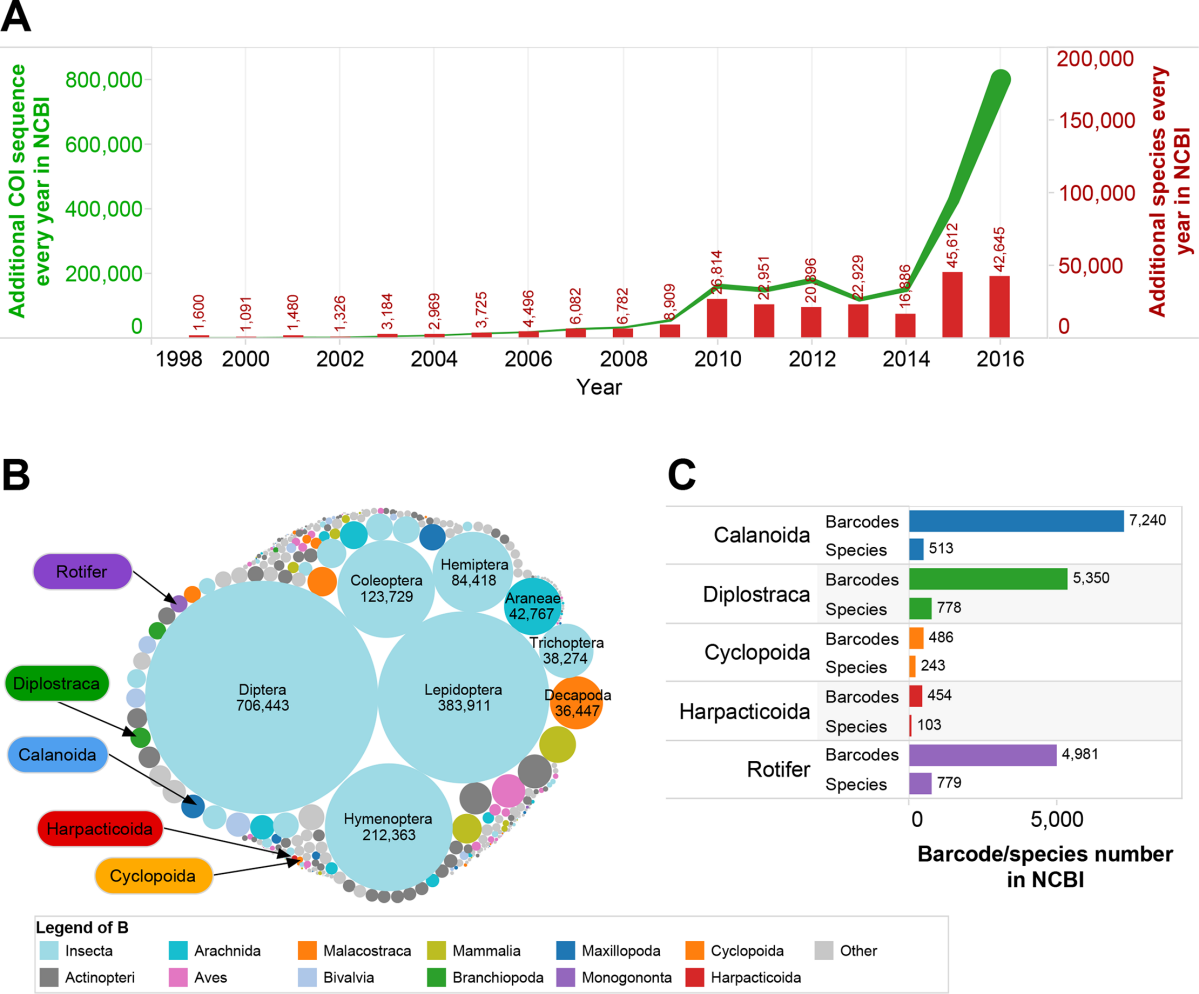
Composition of *COI* barcode sequence in NCBI Genbank. *COI* sequences were downloaded from NCBI Genbank with keyword “COI”. (A): Growth trend of *COI* sequences. (B): Taxa composition of *COI* sequences. (C): Composition of zooplankton *COI* sequences.

Here we developed a high-throughput sequencing protocol to capture *COI* barcode sequences from zooplankton specimens by Ion Torrent PGM and created an indigenous barcode database from 910 native zooplankton specimens. We used both an indigenous barcode database and NCBI public database (consist of all of the *COI* sequences in NCBI Genbank) to annotate the zooplankton metabarcoding data of Tai Lake (China). The aims of this study were to 1) develop a local species barcode database using a high-throughput sequencing species barcoding protocol (Figs 1 and 2) to evaluate the performance of species annotation of metabarcoding data by the local zooplankton barcode database. (S1 Fig).

## Materials and methods

Ethics statement: There are no specific permissions required for the sampling locations as the monitoring project was performed by the local government. This field study did not involve any endangered or protected species and only zooplankton were were collected.

### NCBI public *COI* reference database

The NCBI public *COI* reference database consisted of all the *COI* sequences downloaded from the NCBI Genbank with the key word “COI”. The composition of the NCBI public *COI* reference database were analyzed by R (3.2.3 version).

### Zooplankton sampling

#### For construction of an indigenous barcode database

Surface water was collected by an organic glass hydrophore at depth of 5 cm and filtered by a plankton net (46-μm mesh) at different locations in Lake Tai basin (S2 Fig). Zooplankton samples were fixed with 90% ethanol on site. In the laboratory, zooplankton were washed three times in deionized water and individually selected and transferred to 96-well plates under a stereoscope. Each well contained a single individual. All organisms were identified to the species level by morphology according to Fauna Sinica [17, 18] which is the most authoritative reference for taxonomic identification in China. There were a few cases where specimens could be identified to genus level or higher, such as Mesocyclops species (S1 Table). Zooplankton were classified into three categories by abundance frequency: abundant (frequency > 1/2 samples), moderate (frequency > 1/3 samples) and rare species (frequency < 1/3 samples) (S1 Table).

#### For metabarcoding analysis

Two samples were collected at each site for metabarcoding analysis and morphological identification, respectively (S2 Fig). The bulk sample was collected by a plankton net (46-μm mesh) and filtering ~ 30 L of lake water (at 5 cm depth). Water samples were filtered through 5-μm microporous filter paper (Millipore, USA) and stored at −20°C.

### Zooplankton DNA isolation and PCR amplification

#### For construction of indigenous barcode database

The *COI* fragments were sequenced by Ion Torrent PGM (Fig 1). DNA was extracted from each zooplankton using the HotShot protocol [19]. The organisms were placed in 0.2-mL tubes, and digested in 30-μL of alkaline lysis buffer (NaOH 25 mM, disodium EDTA 0.2 mM, pH 8.0). The digested samples were incubated at 95°C for 30 min and stored on ice for 3–5 min. A further 30 μL of neutralizing buffer was added to each tube and debris removed by centrifugation. PCR amplification was performed in a final volume of 50 μL, made up of 1 μL of 10 μM of universal forward (GGWACWGGWTGAACWGTWTAYCCYCC) and reverse (TAAACTTCAGGGTGACCAAARAAYCA) primers [7], 37.8 μL of ultrapure water, 5 μL of 10×PCR High Fidelity PCR buffer, 2 μL of MgSO4 (50 mM), 1 μL of dNTP mix (10 mM), 0.2 μL of Platinum Taq DNA polymerase, and 2 μL of DNA template (Invitrogen, USA).

PCRs were performed in 96-well plates using a SureCycler 8800 thermal cycler (Agilent Technologies, USA). Because of the high level of degeneracy of primers, a “touchdown” PCR profile was used to minimize the non-specific amplification. PCR was conducted for 16 initial cycles as follows: denaturation for 10s at 95°C, annealing for 30s at 62°C (-1°C per cycle), and extension for 60s at 72°C, followed by 25 cycles at an annealing temperature of 46°C. The final extension was performed at 72°C for 10 min. A negative control reaction with no DNA template was included. PCR products were detected on a 2% agarose gel, and the gel fragments were purified using the MinElute gel extraction kit (Qiagen, CA, USA). The gel-purified PCR products were quantified using the Qubit dsDNA HS assay kits (Invitrogen, USA), and the final concentration was adjusted to 10 ng/μL using molecular grade water.

#### For metabarcoding analysis

The E.Z.N.A. water DNA kit (Omega, USA) was used to isolate zooplankton DNA trapped on the 5-μm filter paper (Millipore, USA). The samples were homogenized by the MoBio Vortex-Genie2 (MoBio Laboratories Inc., CA, USA) with glass beads. The PCR primers and programs used in indigenous barcode database experiment were also used for zooplankton metabarcoding analysis.

### Ion Torrent PGM sequencing

To ensure a homogeneous number of sequencing reads from each specimen, PCR amplicons were mixed in equal concentrations (10 ng/μL) in an equimolar pool. Total 100 ng of amplicon was used in the end-repair and ligation of the adaptors using the Ion Plus fragment library kit (Life Technologies, USA) according to the manufacturer’s protocols. The end-repaired and ligated adaptor DNA was purified with the Agencourt AMPure XP kit (Beckman Coulter, Germany) to eliminate primer dimers and PCR artifacts < 100 bp. The purified amplicon library was assessed for region size distribution and DNA concentration using an Agilent 2100 bioanalyzer (Agilent Technologies, USA). The quantified amplicon libraries were sequenced using the Ion Torrent PGM (Life Technologies, USA).

### Bioinformatics analysis

#### Indigenous barcode database

The ION Torrent server auto-sorts the sequences into different groups based on the library barcode and generates a FASTQ file. The Fastx toolkits and Bio-python were used to reverse complement the FASTQ file and to convert the FASTQ to FASTA [20]. We used the QIIME (Quantitative Insights into Microbial Ecology v1.8.0) platform [21] to filter low-quality reads and to discard reads with more than two mismatches in primer sequence. Chimeras were identified and removed by UCHIME [22]. The above steps were completed using the Bio-Linux 8 system, which integrates all of the above-mentioned tools [23]. Short reads (< 200 bp) were filtered using the “Biostrings” package in R with the Bioconductor environment [24]. The high quality, correctly encoded sequences were clustered into different group by the sequence similarity and using the BLASTX to determine the *COI* barcode sequence. The represented sequences of each species were submitted to NCBI Genbank with the accession no. KY091149- KY091230.

#### Metabarcoding analysis

Sequence pre-treatment (de-nosing, quality trimming, length trimming and chimeric check) were performed following the method in the indigenous barcode database. OTUs were clustered following the UPARSE pipeline [25]. For each OTU, a representative sequence was chosen and the Statistical Assignment Package (SAP) was used to assign the representative sequence to a taxonomic group with 95% cutoff value [26] against reference database (NCBI Genbank database and indigenous species database).

### Genetic distances and tree diagram

The Kimura two-parameter (K2P) distance model was used to calculate genetic divergences of zooplanktons [27]. All sequences from one species were used to calculate the intraspecific genetic distances. A tree diagram was constructed using the neighbor-joining (NJ) method, which provided a graphical representation of the patterns of *COI* divergences [28]. The NJ tree was constructed from 87 sequences (one sequence per species) using MEGA 6 software [29].

## Results

### *COI* reference database form NCBI Genbank

There were 2,186,026 *COI* sequences downloaded from NCBI Genbank (up to 2016–11). These sequences belong to 240,451 taxa (Fig 2A). More than half (56.3%) of the *COI* sequences were released in 2015 and 2016 (428,978 and 802,699 new *COI* sequences were released in 2015 and 2016, respectively). More than one third of taxa (36.7%) were released in 2015 and 2016 (45,612 and 42, 645 new taxa were released in 2015 and 2016, respectively). Most of the *COI* sequences in Genbank were insect sequences and only 0.85% (18,511) of them were zooplankton sequences. Calanoida, cladocera and rotifer had 7240, 5350 and 4981 *COI* sequences, respectively, belonging to 513, 778 and 779 species, respectively (Fig 2B & 2C). Only 486 and 454 sequences were cyclopoida and Harpacticoida, respectively.

### *COI* reference database of indigenous species

**Fig 3:**
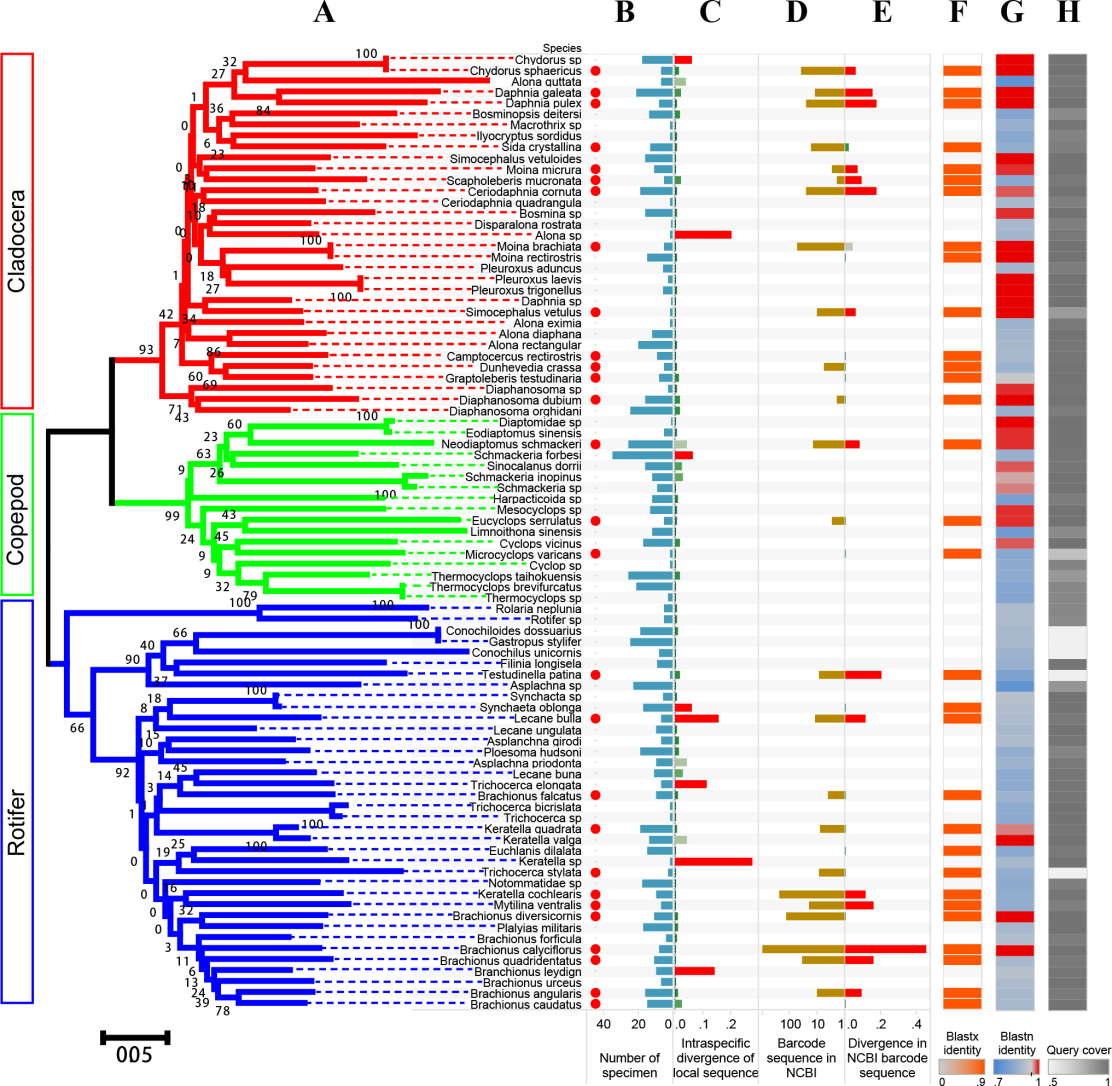
Zooplankton species in the indigenous barcode database of Lake Tai. (A) A tree diagram of representative sequences for each species. Distance was measured as the number of base substitutions per site, based on the Kimura two-parameter (K2P) method. One thousand bootstrap trials were run using the neighbor-joining algorithm of the Mega 6.0 program. (B) Number of specimens of each species; red dot means that the species have barcode sequence in NCBI Genbank. (C) Intraspecific divergence based on the indigenous sequences. (D) *COI* sequences in NCBI Genbank. (E) Intraspecific divergence based on the NCBI Genbank sequences. (F) Similarity of indigenous DNA sequence against NCBI Genbank using Blastx. (G) Similarity of indigenous amino acid sequence against NCBI Genbank using Blastn. (H) Converge of indigenous DNA sequence against NCBI Genbank using Blastn.

The 910 zooplankton specimens, belonging to 87 morphospecies (33 cladocera, 17 copepods, and 37 rotifers), were used to construct the indigenous barcode database (Fig 3). The *COI* sequences were divided into three groups (cladocerans, copepods, and rotifers) in the phylogenetic tree (Fig 3A). The intraspecific divergence of most species was < 5%, except *B*. *leydign* (Rotifer, 14.3%), *T*. *elongate* (Rotifer, 11.5%), *L*. *bulla* (Rotifer, 15.9%), *S*. *oblonga* (Rotifer, 5.95%) and *S*. *forbesi* (Copepod, 6.5%) (Fig 3B & 3C). Most zooplankton in the present study were discriminated by the *COI* sequences, except *Moina brachiate* against *Moina rectirostris* (Cladocera), *Pleuroxus laevis* against *Pleuroxus trigonellus* (Cladocera) and *Conochiloides dossuarius* against *Gastropus stylifer* (Rotifer). There were 28 species with *COI* sequences in NCBI Genbank and 14 of them had intraspecific divergence > 5% based on the NCBI sequences (Fig 3D & 3E). The amino acid sequences of indigenous species were very similar to the corresponding sequences in NCBI Genbank, but the nucleotide sequences between were quite different (Fig 3F, 3G & 3H).

### Species identified by morphological method

In Lake Tai, 76 zooplanktons were identified by the morphologic identification. All of 9 abundant species, 9 of 12 moderate species and 30 of 55 rare species had barcode sequences in the indigenous database (S1 Table). Twenty-four of 76 species had barcode sequences in the NCBI Genbank. Only 3 of 24 species (*Brachionus calyciflorus*, *Keratella cochleari* and *Brachionus diversicornis*) had > 100 *COI* sequences in the NCBI Genbank (S3 Fig).

### Taxonomic assignment of NGS data between NCBI and indigenous database

**Fig 4:**
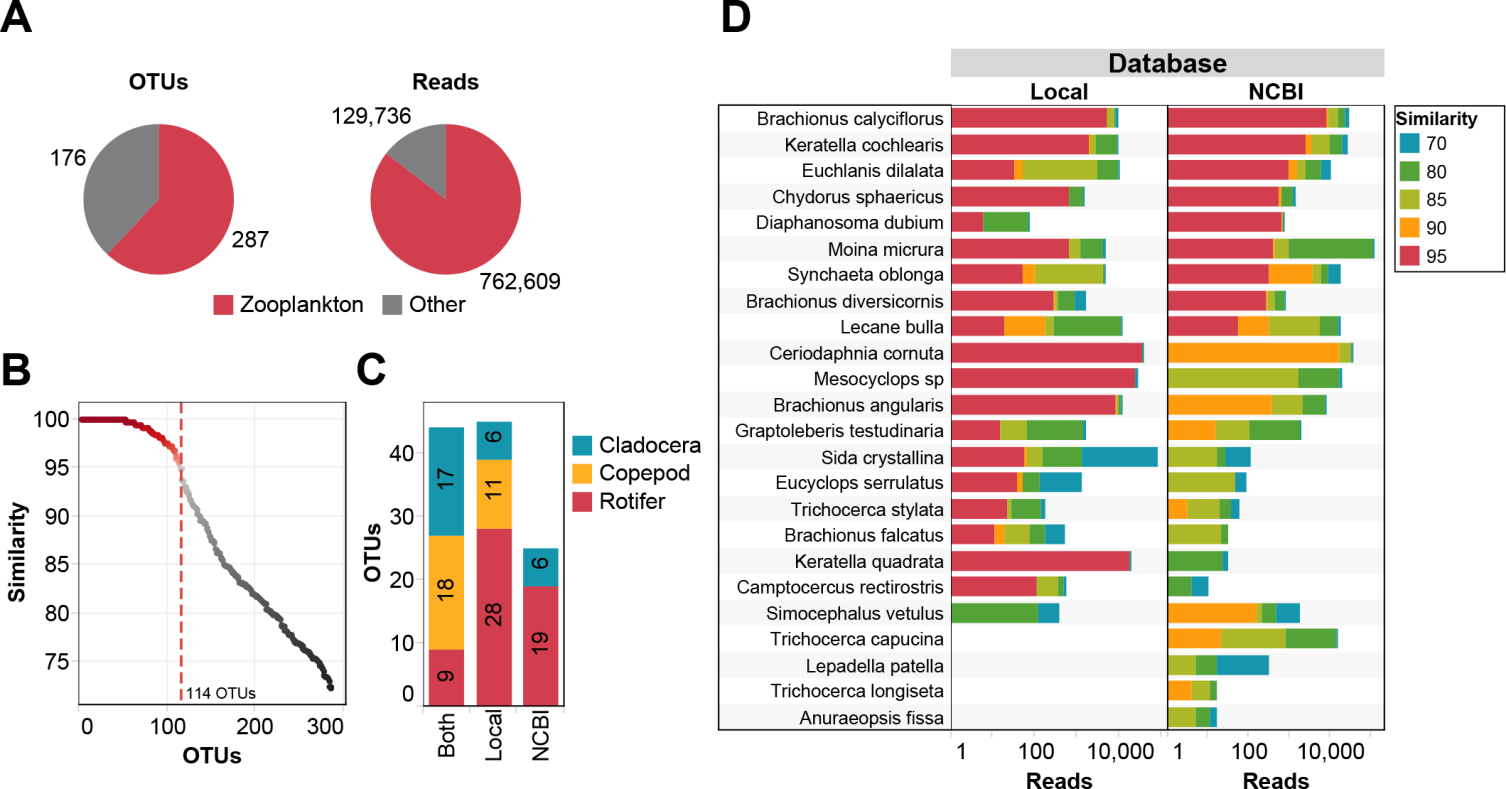
Taxonomic assignment of NGS data. (A) Numbers of zooplankton OTUs and sequences in the NGS data. (B) Distribution of sequence similarity of OTUs against database (both indigenous and NCBI Genbank database). (C) Number of OTUs annotated by indigenous database and/or NCBI Genbank database. “Local” means the OTUs annotated by the indigenous database and “NCBI” means the OTUs annotated by NCBI Genbank. (D) Comparison of NGS data annotated by indigenous database and NCBI Genbank database. Only 24 species that have barcode sequence in NCBI Genbank were showed.

After pre-treatment, 892,345 *COI* sequences were recovered by high-throughput sequencing. These sequences were clustered to 463 unique OTUs, among which 287 OTUs (represented 762,609 reads) belong to zooplankton (Fig 4A). Forty-four zooplankton OTUs were assigned to species level (similarity > 95%, alignment length > 100 bp) by both the indigenous species and NCBI Genbank databases. Twenty-five and 45 OTUs were assigned to the species level only using the NCBI Genbank database and indigenous species database, respectively (Fig 4C).

**Fig 5:**
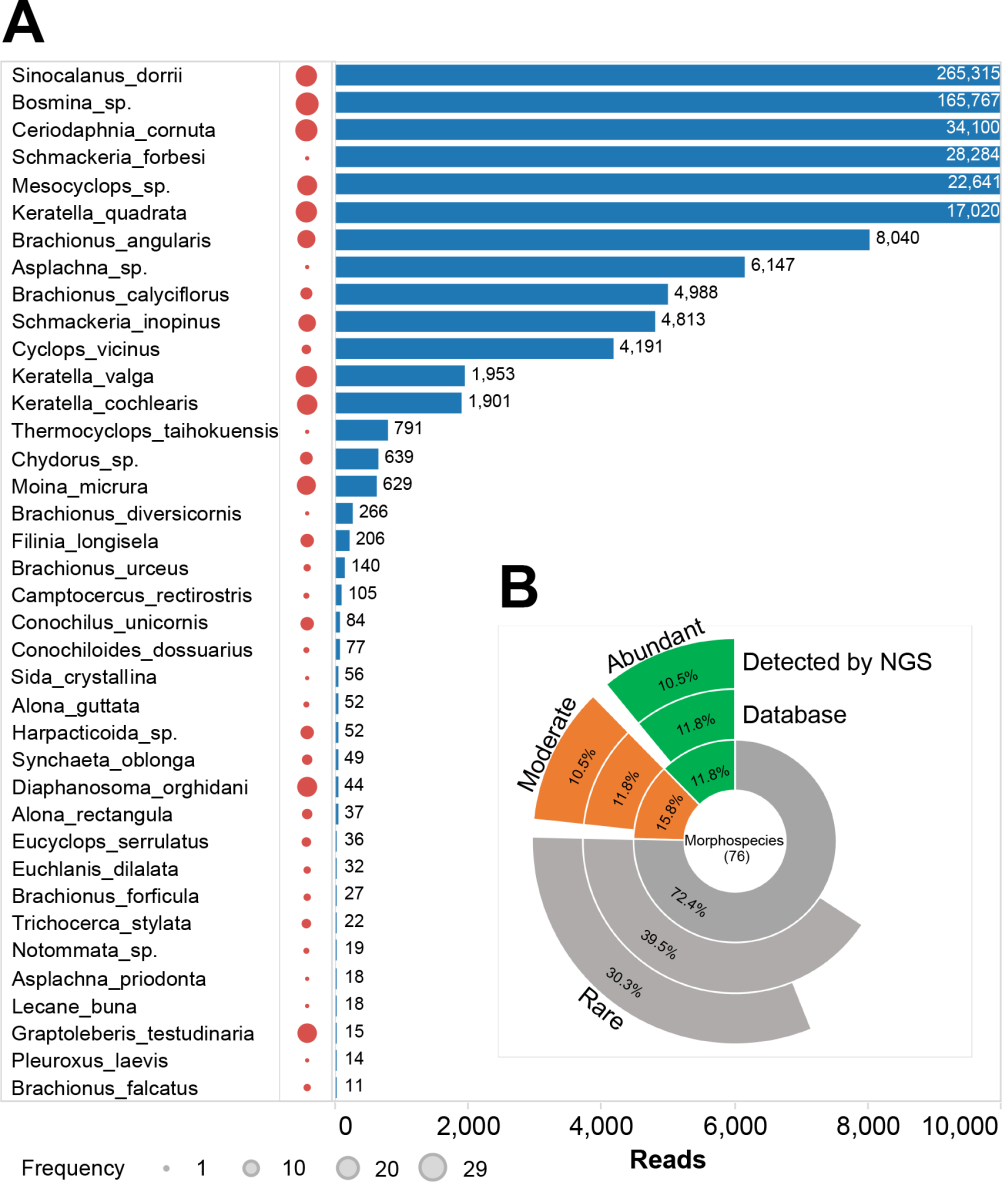
Species identified by metabarcoding analysis. The size of red dots indicated the frequency of each species that detected by morphology method (A) Reads number of each species in metabarcoding data. (B) The internal arcs indicate the species found in morphological analysis. The middle arcs indicate the species that have barcode sequences in indigenous species database. The external arcs indicate which species were detected by metabarcoding. Abundant (detected frequency > 1/2), moderate (detected frequency > 1/3) and rare (detected frequency < 1/3).

Thirty-nine of 76 morphological species were detected by the metabarcoding (Fig 5). Of the 39 zooplankton identified, nine were identified by both the indigenous database and NCBI Genbank database (Fig 4D). The remaining 30 species were only identified by the indigenous database (similarity > 95%).

Copepod *Sinocalanus dorrii* and Cladocera *Bosmina sp*. had high reads numbers and represented 265, 315 (35.2%) and 165, 767 (22.0%) reads, respectively. Eight abundant species, 8 moderate species, and 23 rare species were identified by metabarcoding (Fig 5A & 5B). Both *Schmackeria forbesi* (Copepod) and *Asplachna sp*. (Rotifer) species had more than 5000 reads, although they had a low occurrence frequency using morphology identification. Cladocera *Graptoleberis testudinaria* and *Diaphanosoma orghidani* contained 44 and 15 sequences (Fig 5A).

### Comparison between metabarcoding and morphological monitoring

**Fig 6:**
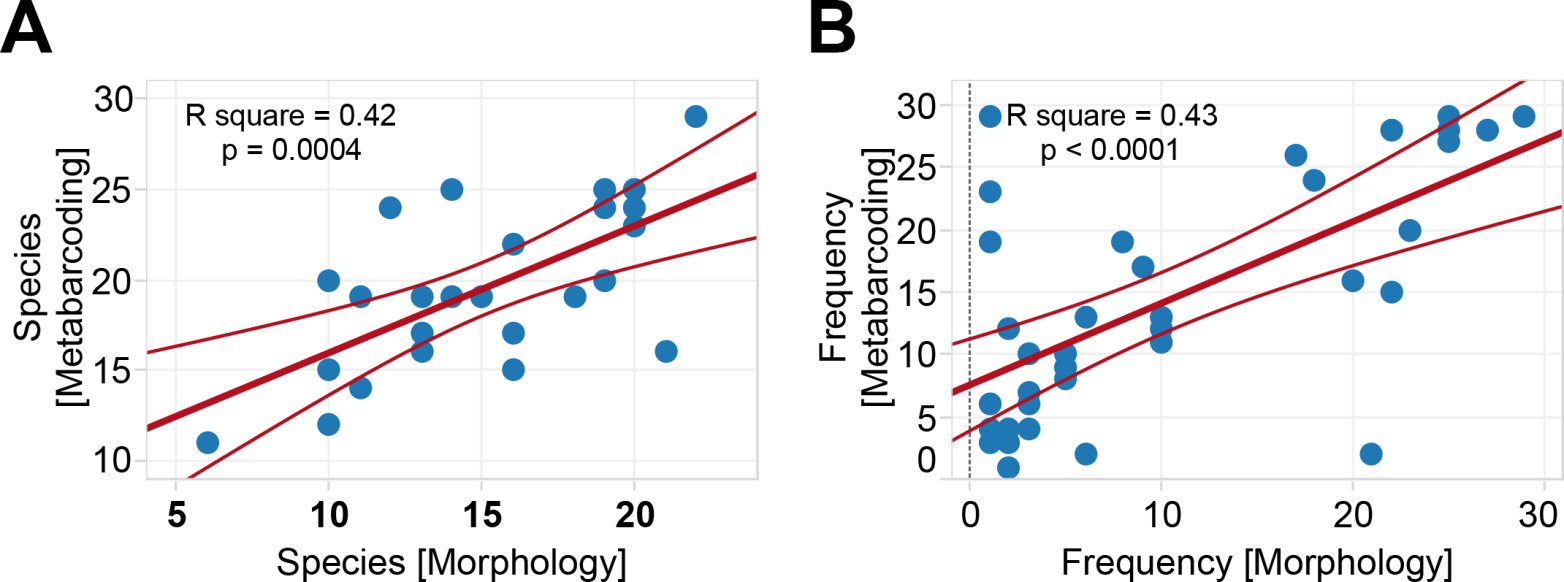
Comparison of zooplankton identification in water samples between metabarcoding and morphology approaches. (A) Species number. (B) Frequency detected. The R2 and p-value are indicated for each regression axis.

Morphology data demonstrated that Copepod *S*. *dorrii* and *Mesocyclops sp*., Cladocera *B*. *sp*. and *Ceriodaphnia cornuta*, and Rotifer *Keratella quadrata* were the dominant zooplankton in Lake Tai. These species also represented a greater reads number and had a higher detected frequency by the metabarcoding than other zooplankton (Fig 5A). Cladocera *Limnoithona sinensis* was not identified by metabarcoding, although it had a high frequency in the morphology data. Copepod *S*. *forbesi* and *Thermocyclops taihokuensis* and Rotifer *B*. *diversicorni* showed high detection frequency in metabarcoding data, but had low detection rates in the morphological data (S4 Fig). The number of species detected by metabarcoding in each sample was positively correlated (R^2^ = 0.42, *p* = 0.0004) with that by morphological identification (Fig 6A). Furthermore, the frequency of species in metabarcoding also positively correlated (R^2^ = 0.43, *p* < 0.0001) with morphology identification (Fig 6B).

## Discussion

In the present study, we constructed an indigenous *COI* barcode database of zooplankton from the Tai Lake basin of Eastern China, and then compared indigenous database and NCBI Genbank in the annotation of the zooplankton metabarcoding. The indigenous database improved the taxonomic assignment of metabarcoding of zooplankton. Furthermore, the similarity of species identification of the common species between microscopic and metabarcoding was confirmed. First, most zooplankton (81%) which had barcode sequences in the indigenous database were identified by metabarcoding. Second, the species number observed by metabarcoding was positively correlated with that identified by microscope. Finally, the distributions of common zooplankton are highly similar between the two methods. These results are not new observations, but confirm that the *COI* barcode can successful identify most species of zooplanktons and metabarcoding is well suited for biodiversity monitoring of zooplankton. Although the metabarcoding monitoring of zooplankton is promising, there is still an opportunity to reduce the divergences between molecular and morphological monitoring by addressing the current limitations of metabarcoding. Some technical biases related to DNA extraction, PCR conditions, primer specificity, library preparation and bioinformatics have been extensively discussed in previous studies [30–32]. Below, limitations of (1) incompleteness, inaccuracy and the high divergence of zooplankton databases; and (2) inefficiency of barcode sequences captured by Sanger sequencing are discussed.

### Incompleteness, inaccuracy and high divergence of zooplankton databases

Metabarcoding-based species identification requires taxonomically complete and geographically comprehensive reference databases of DNA sequences for each species [33, 34]. Incompleteness and inaccuracy of databases are commonly believed to be the main hindrance to the use of metabarcoding [35]. Although *COI* sequences are growing fast, the identification of zooplankton by only relying on the NCBI Genbank is inefficient. This is not only because of database incompleteness, but also due to the high divergence of zooplankton [36, 37]. Only 0.85% of the *COI* sequences belong to zooplankton in NCBI Genbank. Here, 24 out of 76 zooplanktons identified by morphology have records in Genbank but only nine of them were identified to the species level by NCBI Genbank. The sequences of NCBI Genbank come from all over the world. These sequences show high levels of intraspecific divergence of most zooplankton species, suggesting a geographical difference (Fig 1E). Furthermore, indigenous species sequences also show a high level of divergence compared with the sequences from NCBI (Fig 1G). This explains why some species cannot be assigned to the species level by NCBI. It is well known that *COI* fragment appears to possess a greater range of phylogenetic signal than any other mitochondrial and nuclear gene [38]. In fact, the evolution of *COI* is rapid enough to allow the discrimination of not only closely allied species, but also phylo-geographic groups within a single species [39, 40]. Zooplankton, such as rotifer, often have complex life cycles, high dispersal capacities and rapid local adaptations, which may facilitate interspecific gene flow and intraspecific divergence [41, 42]. Previous studies has discussed the high divergence and cryptic species in zooplankton [37, 43, 44]. For example, up to 15 *COI* genetic groups were found in one of the common Rotifer, *B*. *calyciflorus*, among 22 lakes in Netherlands [45]. This species also had a high intraspecific divergence in China [46]. Another possible reason for the high divergence of zooplankton in the NCBI database is the misidentification of zooplankton; especially for rotifers where taxonomy remains unclear [47] with few taxonomist experts [48]. In addition, the ability to discriminate between species on the basis of morphological characteristics is limited by the high level of phenotypic variation [13]. Different morphological variants have often been described as different species, subspecies, or forms [49]. Overall, incompleteness, inaccuracy and high divergence of zooplankton reference databases is a challenge for studying zooplankton metabarcoding. This can be addressed by the barcode database of indigenous species, especially for the metabarcoding based on the mitochondrial *COI* region.

### Inefficiency of barcode sequence captured by sanger sequencing

The high-throughput sequencing platform improves the DNA barcode capture from zooplankton. Although an indigenous species database is important for metabarcoding, capturing the barcode sequence of zooplankton was inefficient by Sanger sequencing. We attempted to construct a taxonomic DNA barcode library of a large number of zooplankton samples by the high-throughput sequencing platform. The results demonstrated the potential of high-throughput sequencing as an effective method to capture barcode sequences of zooplankton.

The shortage of DNA barcode sequences in public databases for small body organisms such as zooplankton, may be due to the limitation of conventional approaches of generating barcode sequence, which is by PCR amplification and Sanger sequencing [50]. The low yield and low quality genomic DNA of single zooplankton specimen leads to low-efficiency PCR and low successful rates of Sanger sequencing [5, 51]. In addition, insufficient amplification due to primer specificity, co-amplification of non-target amplicons also causes barcoding failures [5]. For example, in addition to the target barcode sequence, sequences from *Wolbachia* were also detected in some specimens of insect Lepidoptera [5]. The presence of *Wolbachia* [52, 53], pseudogenes and heteroplasmy in public *COI* sequence databases could compromise the identification of DNA barcode specimens [14, 15].

These problems can be solved using high-throughput sequencing. First, high-throughput sequencing only requires a small amount of DNA (e.g. 100 pM for ION torrent PGM) to sequence. In addition, high-throughput sequencing can generate multiple sequences for a single specimen. The non-target sequences can be identified by examining the sequence similarity and subsequently removed and improve the efficiency of recover DNA sequences in a single attempt [5]. Although the Sanger sequencing remains the major way for barcode sequence capture, the low cost and high-throughput of high-throughput sequencing platform will enhance and accelerate the indigenous database construction of zooplankton [16].

## Conclusion

Building up indigenous databases significantly improved the analysis of species-level zooplankton biodiversity by metabarcoding. Although NCBI Genbank contain a large number of *COI* sequences, the contributions of NCBI Genbank to the identification of zooplankton in metabarcoding data are limited. The high-throughput sequencing platform enhanced the DNA barcode capture from single zooplankton specimens and the barcode database of indigenous species significantly improved the taxonomic assignment of metabarcoding data.

## Additional information

The raw sequences of metabatcoding were submitted to NCBI Sequence Read Archive (SRR5202370).

## Supporting information

S1 Table Zooplankton identified by morphological method. “√” means the species have barcode sequence in indigenous database or NCBI Genbank databse. “yes”means the species can be identified by indigenous database or NCBI Genbank.
(DOCX)

S1 Fig The workflow of the present study. (TIF)

S2 Fig Sampling sites in the present study. The sampling sites for indigenous barcode database were indicated by black dots. The sampling sites for zooplankton metabarcoding analysis were indicated by green dots.
(TIF)

S3 Fig The coverage of zooplankton in Taihu Lake by NCBI Genbank. (A): the number of species. (B): the number of *COI* sequence.
(TIF)

S4 Fig Detection of zooplankton species for metabarcoding and morphologic identification and their equivalency across samples. Green indicates presence for metabarcoding, red indicates presence for morphologic identification and white indicates not detected. For the equivalency, black indicates consistency of detection (presence or absence) of the same sample by both methods, white indicates inconsistency of specie detection.
(TIF)

## References

[1] Steinberg DK, Van Mooy BA, Buesseler KO, Boyd PW, Kobari T, Karl DM. Bacterial vs. zooplankton control of sinking particle flux in the ocean's twilight zone. Limnology and Oceanography. 2008;53(4):1327–38.

[2] Ferdous Z, Muktadir A. A Review: Potentiality of Zooplankton as Bioindicator. American Journal of Applied Sciences. 2009;6(10):1815–9.

[3] Machida RJ, Hashiguchi Y, Nishida M, Nishida S. Zooplankton diversity analysis through single-gene sequencing of a community sample. Bmc Genomics. 2009;10(1):438. 19758460 10.1186/1471-2164-10-438 PMC2751789

[4] Lindeque PK, Parry HE, Harmer RA, Somerfield PJ, Atkinson A. Next generation sequencing reveals the hidden diversity of zooplankton assemblages. PloS one. 2013;8(11):e81327 doi: 10.1371/journal.pone.0081327 24244737

[5] Shokralla S, Gibson JF, Nikbakht H, Janzen DH, Hallwachs W, Hajibabaei M. Next‐generation DNA barcoding: using next‐generation sequencing to enhance and accelerate DNA barcode capture from single specimens. Molecular ecology resources. 2014;14(5):892–901. doi: 10.1111/1755-0998.12236 24641208 10.1111/1755-0998.12236 PMC4276293

[6] Hajibabaei M, Shokralla S, Zhou X, Singer GA, Baird DJ. Environmental barcoding: a next-generation sequencing approach for biomonitoring applications using river benthos. PLoS one. 2011;6(4):e17497 doi: 10.1371/journal.pone.0017497 21533287 10.1371/journal.pone.0017497 PMC3076369

[7] Leray M, Yang JY, Meyer CP, Mills SC, Agudelo N, Ranwez V, et al A new versatile primer set targeting a short fragment of the mitochondrial COI region for metabarcoding metazoan diversity: application for characterizing coral reef fish gut contents. Front Zool. 2013;10(1):34. 23767809 10.1186/1742-9994-10-34 PMC3686579

[8] Leray M, Boehm J, Mills SC, Meyer C. Moorea BIOCODE barcode library as a tool for understanding predator–prey interactions: insights into the diet of common predatory coral reef fishes. Coral reefs. 2012;31(2):383–8.

[9] Frolov S, Kudela RM, Bellingham JG. Monitoring of harmful algal blooms in the era of diminishing resources: a case study of the US West Coast. Harmful Algae. 2013;21:1–12.

[10] Bourlat SJ, Borja A, Gilbert J, Taylor MI, Davies N, Weisberg SB, et al Genomics in marine monitoring: new opportunities for assessing marine health status. Mar Pollut Bull. 2013;74(1):19–31. doi: 10.1016/j.marpolbul.2013.05.042 23806673 10.1016/j.marpolbul.2013.05.042

[11] Coissac E, Riaz T, Puillandre N. Bioinformatic challenges for DNA metabarcoding of plants and animals. Molecular Ecology. 2012;21(8):1834–47. doi: 10.1111/j.1365-294X.2012.05550.x 22486822 10.1111/j.1365-294X.2012.05550.x

[12] Leray M, Knowlton N. DNA barcoding and metabarcoding of standardized samples reveal patterns of marine benthic diversity. P Natl Acad Sci USA. 2015;112(7):2076–81. 10.1073/pnas.1424997112 PMC4343139 25646458

[13] García-Morales A, Elías-Gutiérrez M. DNA barcoding of freshwater Rotifera in Mexico: evidence of cryptic speciation in common rotifers. Molecular ecology resources. 2013;13(6):1097–107. doi: 10.1111/1755-0998.12080 23433240 10.1111/1755-0998.12080

[14] Song H, Buhay JE, Whiting MF, Crandall KA. Many species in one: DNA barcoding overestimates the number of species when nuclear mitochondrial pseudogenes are coamplified. P Natl Acad Sci USA. 2008;105(36):13486–91. 10.1073/pnas.0803076105 PMC2527351 18757756

[15] Smith MA, Bertrand C, Crosby K, Eveleigh ES, Fernandez-Triana J, Fisher BL, et al Wolbachia and DNA barcoding insects: patterns, potential, and problems. PloS one. 2012;7(5):e36514 doi: 10.1371/journal.pone.0036514 22567162 10.1371/journal.pone.0036514 PMC3342236

[16] Shokralla S, Porter TM, Gibson JF, Dobosz R, Janzen DH, Hallwachs W, et al Massively parallel multiplex DNA sequencing for specimen identification using an Illumina MiSeq platform. Scientific Reports. 2015;5:9687 doi: 10.1038/srep09687 25884109 10.1038/srep09687 PMC4401116

[17] Chiang Sieh-chih DN-s. Fauna Sinica: Crustacea Freshwater Cladocera. Sinica FECA, editor. Peking China: Science Press; 1979.

[18] Shen Chia-jui TA-y, Zhang Chong-zhou, Li Zhi-ying, Song Da-xiang, Song Yu-zhi, Chen Guo-xiao. Fauna Sinica: Crustacea Freshwater Copepoda. Chia-jui S, editor. Peking, China: Science Press; 1979.

[19] Montero‐Pau J, Gómez A, Muñoz J. Application of an inexpensive and high‐throughput genomic DNA extraction method for the molecular ecology of zooplanktonic diapausing eggs. Limnology and Oceanography: Methods. 2008;6(6):218–22.

[20] Cock PJ, Antao T, Chang JT, Chapman BA, Cox CJ, Dalke A, et al Biopython: freely available Python tools for computational molecular biology and bioinformatics. Bioinformatics. 2009;25(11):1422–3. doi: 10.1093/bioinformatics/btp163 19304878 10.1093/bioinformatics/btp163 PMC2682512

[21] Caporaso JG, Kuczynski J, Stombaugh J, Bittinger K, Bushman FD, Costello EK, et al QIIME allows analysis of high-throughput community sequencing data. Nature methods. 2010;7(5):335–6. doi: 10.1038/nmeth.f.303 20383131 10.1038/nmeth.f.303 PMC3156573

[22] Edgar RC, Haas BJ, Clemente JC, Quince C, Knight R. UCHIME improves sensitivity and speed of chimera detection. Bioinformatics. 2011;27(16):2194–200. doi: 10.1093/bioinformatics/btr381 21700674 10.1093/bioinformatics/btr381 PMC3150044

[23] Field D, Tiwari B, Booth T, Houten S, Swan D, Bertrand N, et al Open software for biologists: from famine to feast. Nature biotechnology. 2006;24(7):801–3. doi: 10.1038/nbt0706-801 16841067 10.1038/nbt0706-801

[24] Gentleman RC, Carey VJ, Bates DM, Bolstad B, Dettling M, Dudoit S, et al Bioconductor: open software development for computational biology and bioinformatics. Genome Biol. 2004;5(10):R80 PubMed PMID: WOS:000224243400013. doi: 10.1186/gb-2004-5-10-r80 15461798 10.1186/gb-2004-5-10-r80 PMC545600

[25] Edgar RC. UPARSE: highly accurate OTU sequences from microbial amplicon reads. Nature Methods. 2013;10(10):996–8. doi: 10.1038/nmeth.2604 PubMed PMID: WOS:000325073800023. 23955772 10.1038/nmeth.2604

[26] Munch K, Boomsma W, Huelsenbeck JP, Willerslev E, Nielsen R. Statistical Assignment of DNA Sequences Using Bayesian Phylogenetics. Systematic Biology. 2008;57(5):750–7. doi: 10.1080/10635150802422316 PubMed PMID: WOS:000259995600007. 18853361 10.1080/10635150802422316

[27] Kimura M. A simple method for estimating evolutionary rates of base substitutions through comparative studies of nucleotide sequences. J Mol Evol. 1980;16(2):111–20. 7463489 10.1007/BF01731581

[28] Saitou N, Nei M. The neighbor-joining method: a new method for reconstructing phylogenetic trees. Mol Biol Evol. 1987;4(4):406–25. 3447015 10.1093/oxfordjournals.molbev.a040454

[29] Tamura K, Stecher G, Peterson D, Filipski A, Kumar S. MEGA6: molecular evolutionary genetics analysis version 6.0. Mol Biol Evol. 2013;30(12):2725–9. doi: 10.1093/molbev/mst197 24132122 10.1093/molbev/mst197 PMC3840312

[30] Lee CK, Herbold CW, Polson SW, Wommack KE, Williamson SJ, Mcdonald IR, et al Groundtruthing Next-Gen Sequencing for Microbial Ecology-Biases and Errors in Community Structure Estimates from PCR Amplicon Pyrosequencing. PLOS ONE. 2012;7(9):1–12. 10.1371/journal.pone.0044224 PMC3435322 22970184

[31] Esling P, Lejzerowicz F, Pawlowski J. Accurate multiplexing and filtering for high-throughput amplicon-sequencing. Nucleic Acids Research. 2015;43(5):2513–24. doi: 10.1093/nar/gkv107 25690897 10.1093/nar/gkv107 PMC4357712

[32] Sun C, Zhao YL, Li H, Dong Y, Macisaac HJ, Zhan A. Unreliable quantitation of species abundance based on high-throughput sequencing data of zooplankton communities. Aquatic Biology. 2015;24(1):9–15.

[33] Visco JA, Apothéloz-Perret-Gentil L, Cordonier A, Esling P, Pillet L, Pawlowski J. Environmental Monitoring: Inferring the Diatom Index from Next-Generation Sequencing Data. Environmental Science & Technology. 2015;49(13):7597–605. 26052741 10.1021/es506158m

[34] Blancobercial L, Cornils A, Copley N, Bucklin A. DNA barcoding of marine copepods: assessment of analytical approaches to species identification. 2014;6(6):S122. 10.1371/currents.tol.cdf8b74881f87e3b01d56b43791626d2 PMC4073882 24987576

[35] Bucklin A, Lindeque PK, Rodriguez-Ezpeleta N, Albaina A, Lehtiniemi M. Metabarcoding of marine zooplankton: prospects, progress and pitfalls. Journal of Plankton Research. 2016;38(3):393–400. doi: 10.1093/plankt/fbw023

[36] Gómez A, Serra M, Carvalho GR, Lunt DH. Speciation in ancient cryptic species complexes: evidence from the molecular phylogeny of Brachionus plicatilis (Rotifera). Evolution. 2002;56(7):1431–44. 12206243 10.1111/j.0014-3820.2002.tb01455.x

[37] Suatoni E, Vicario S, Rice S, Snell T, Caccone A. An analysis of species boundaries and biogeographic patterns in a cryptic species complex: the rotifer—Brachionus plicatilis. Molecular Phylogenetics & Evolution. 2006;41(1):86–98. 16815046 10.1016/j.ympev.2006.04.025

[38] Hebert PD, Cywinska A, Ball SL, Dewaard JR. Biological identifications through DNA barcodes. Proceedings Biological Sciences. 2003;270(1512):313–21. doi: 10.1098/rspb.2002.2218 12614582 10.1098/rspb.2002.2218 PMC1691236

[39] Cox AJ, Hebert PD. Colonization, extinction, and phylogeographic patterning in a freshwater crustacean. Molecular Ecology. 2001;10(2):371–86. 11298952 10.1046/j.1365-294x.2001.01188.x

[40] Wares JP, Cunningham CW. Phylogeography and historical ecology of the North Atlantic intertidal. Evolution. 2001;55(12):2455–69. 11831661 10.1111/j.0014-3820.2001.tb00760.x

[41] Gómez A, Adcock GJ, Lunt DH, Carvalho GR. The interplay between colonization history and gene flow in passively dispersing zooplankton: microsatellite analysis of rotifer resting egg banks. Journal of Evolutionary Biology. 2002;15(1):158–71.

[42] Cristescu ME, Constantin A, Bock DG, Cáceres CE, Crease TJ. Speciation with gene flow and the genetics of habitat transitions. Molecular Ecology. 2012;21(6):1411–22. doi: 10.1111/j.1365-294X.2011.05465.x 22269101 10.1111/j.1365-294X.2011.05465.x

[43] Gilbert JJ, Walsh EJ. Brachionus calyciflorus is a Species Complex: Mating Behavior and Genetic Differentiation Among Four Geographically Isolated Strains. Hydrobiologia. 2005;546(1):257–65.

[44] Elias-Gutierrez M, Jerónimo FM, Ivanova NV, Valdez-Moreno M, Hebert PDN. DNA barcodes for Cladocera and Copepoda from Mexico and Guatemala, highlights and new discoveries. Zootaxa. 2008;42(1839):1–42.

[45] Papakostas S, Michaloudi E, Proios K, Brehm M, Verhage L, Rota J, et al Integrative Taxonomy Recognizes Evolutionary Units Despite Widespread Mitonuclear Discordance: Evidence from a Rotifer Cryptic Species Complex. Systematic Biology. 2016;65(3):508–24. doi: 10.1093/sysbio/syw016 26880148 10.1093/sysbio/syw016

[46] Xiang XL, Xi YL, Wen XL, Zhang G, Wang JX, Hu K. Patterns and processes in the genetic differentiation of the Brachionus calyciflorus complex, a passively dispersing freshwater zooplankton. Molecular Phylogenetics & Evolution. 2011;59(2):386–98. 21335094 10.1016/j.ympev.2011.02.011

[47] Wallace RL. Rotifers: Exquisite Metazoans. Integrative and Comparative Biology. 2002;42(3):660–7. doi: 10.1093/icb/42.3.660 21708762 10.1093/icb/42.3.660

[48] Segers H. Global diversity of rotifers (Phylum Rotifera) in freshwater. Hydrobiologia. 2008;595(1):49–59.

[49] Segers H, De Smet WH. Diversity and endemism in Rotifera: a review, and Keratella Bory de St Vincent. Biodiversity and Conservation. 2008;17(2):303–16.

[50] Sanger F, Nicklen S, Coulson AR. DNA sequencing with chain-terminating inhibitors. P Natl Acad Sci USA. 1977;74(12):5463–7. 10.1073/pnas.74.12.5463 PMC431765 271968

[51] Polz MF, Cavanaugh CM. Bias in template-to-product ratios in multitemplate PCR. Appl Environ Microbiol. 1998;64(10):3724–30. 9758791 10.1128/aem.64.10.3724-3730.1998 PMC106531

[52] Wiwatanaratanabutr I, Grandjean F. Impacts of temperature and crowding on sex ratio, fecundity and Wolbachia infection intensity in the copepod, Mesocyclops thermocyclopoides. Journal of Invertebrate Pathology. 2016;141:18–23. doi: 10.1016/j.jip.2016.10.003 27756651 10.1016/j.jip.2016.10.003

[53] Wiwatanaratanabutr I. Distribution, diversity and density of wolbachial infections in cladocerans and copepods from Thailand. Journal of Invertebrate Pathology. 2013;114(3):341 doi: 10.1016/j.jip.2013.04.014 24080157 10.1016/j.jip.2013.04.014

