# Supplementary material for: Indigenous species barcode database improves the identification of zooplankton
Source: PLoS One. 2017 Oct 4;12(10):e0185697. doi: 10.1371/journal.pone.0185697 (PMC5627919; doi:10.1371/journal.pone.0185697)
Supplement: S1 Table — “√” means the species have barcode sequence in indigenous database or NCBI Genbank databse. “yes”means the species can be identified by indigenous database or NCBI Genbank. (DOCX) [file pone.0185697.s001.docx]

Table S1. Zooplankton identified by morphological method.

| Category | Species | Indigenous database | Identified by indigenous database | NCBI Genbank | Identified by NCBI |
| --- | --- | --- | --- | --- | --- |
| abundant | *Bosmina sp.* | √ | yes |  |  |
| abundant | *Limnoithona sinensis* | √ |  |  |  |
| abundant | *Sinocalanus dorrii* | √ | yes |  |  |
| abundant | *Ceriodaphnia cornuta* | √ | yes | √ |  |
| abundant | *Keratella valga* | √ | yes |  |  |
| abundant | *Keratella cochlearis* | √ | yes | √ | yes |
| abundant | *Schmackeria inopinus* | √ | yes |  |  |
| abundant | *Keratella quadrata* | √ | yes | √ |  |
| abundant | *Conochilus unicornis* | √ | yes |  |  |
| moderate | *Brachionus angularis* | √ | yes | √ |  |
| moderate | *Filinia longisela* | √ | yes |  |  |
| moderate | *Euchlanis dilalata* | √ | yes | √ | yes |
| moderate | *Diaphanosoma orghidani* | √ | yes |  |  |
| moderate | *Moina micrura* | √ | yes | √ | yes |
| moderate | *Polyarthra sp1.* |  |  |  |  |
| moderate | *Pompholyx sulcata* |  |  |  |  |
| moderate | *Graptoleberis testudinaria* | √ | yes | √ |  |
| moderate | *Mesocyclops sp.* | √ | yes |  |  |
| moderate | *Polyarthra sp3.* |  |  |  |  |
| moderate | *Brachionus calyciflorus* | √ | yes | √ | yes |
| moderate | *Gastropus stylifer* | √ |  |  |  |
| rare | *Chydorus sphaericus* | √ |  | √ | yes |
| rare | *Synchaeta oblonga* | √ | yes | √ | yes |
| rare | *Harpacticoida sp.* | √ | yes |  |  |
| rare | *Chydorus sp.* | √ | yes |  |  |
| rare | *Alona rectangula* | √ | yes |  |  |
| rare | *Brachionus falcatus* | √ | yes | √ |  |
| rare | *Conochiloides dossuarius* | √ | yes |  |  |
| rare | *Gastropus minor* |  |  |  |  |
| rare | *Cyclops vicinus* | √ | yes |  |  |
| rare | *Eucyclops serrulatus* | √ | yes | √ |  |
| rare | *Notholon labis* |  |  |  |  |
| rare | *Trichocerca capucina* |  |  | √ |  |
| rare | *Trichocerca sp1.* |  |  |  |  |
| rare | *Trichocerca stylata* | √ | yes | √ |  |
| rare | *Bosminopsis deitersi* | √ |  |  |  |
| rare | *Brachionus forficula* | √ | yes |  |  |
| rare | *Collotheca sp.* |  |  |  |  |
| rare | *Brachionus urceus* | √ | yes |  |  |
| rare | *Trichocerca pusilla* |  |  |  |  |
| rare | *Alona eximia* | √ |  |  |  |
| rare | *Asplachna sp.* | √ | yes |  |  |
| rare | *Keratella ticinensis* |  |  |  |  |
| rare | *Lecane sp1.* |  |  |  |  |
| rare | *Trichocerca sp2.* |  |  |  |  |
| rare | *Alona guttata* | √ | yes |  |  |
| rare | *Ascomorpha saltans* |  |  |  |  |
| rare | *Asplachna priodonta* | √ | yes |  |  |
| rare | *Mesocyclops sp2* |  |  | √ |  |
| rare | *Sida crystallina* | √ | yes | √ |  |
| rare | *Anuraeopsis fissa* |  |  | √ |  |
| rare | *Brachionus diversicornis* | √ | yes | √ | yes |
| rare | *Camptocercus rectirostris* | √ | yes | √ |  |
| rare | *Lecane hornemanni* |  |  |  |  |
| rare | *Lecane lunaris* |  |  |  |  |
| rare | *Pleuroxus trigonellus* | √ |  |  |  |
| rare | *Schmackeria forbesi* | √ | yes |  |  |
| rare | *Simocephalus vetulus* | √ |  | √ |  |
| rare | *Spapholeberis mucronata* |  |  |  |  |
| rare | *Tetramastix sp.* |  |  |  |  |
| rare | *Thermocyclops taihokuensis* | √ | yes |  |  |
| rare | *Trichocerca longiseta* |  |  | √ |  |
| rare | *Daphnia hyalina* |  |  |  |  |
| rare | *Diaphanosoma dubium* | √ | yes | √ | yes |
| rare | *Diaphanosoma sp.* | √ |  |  |  |
| rare | *Filinia sp2.* |  |  |  |  |
| rare | *Lecane buna* | √ | yes | √ | yes |
| rare | *Lepadella patella* |  |  | √ |  |
| rare | *Moina sp.* |  |  |  |  |
| rare | *Notommata sp.* | √ | yes |  |  |
| rare | *Pleuroxus striatus* |  |  |  |  |
| rare | *Acanthocyclops thomasi* |  |  |  |  |
| rare | *Alona costata* |  |  |  |  |
| rare | *Alona diaphana* | √ |  |  |  |
| rare | *Alona sp.* | √ |  |  |  |
| rare | *Pleuroxus laevis* | √ | yes |  |  |
| rare | *Polyarthra sp2.* |  |  |  |  |
