# Supplementary figures and images for: Indigenous species barcode database improves the identification of zooplankton
Source: PLoS One. 2017 Oct 4;12(10):e0185697. doi: 10.1371/journal.pone.0185697 (PMC5627919; doi:10.1371/journal.pone.0185697)

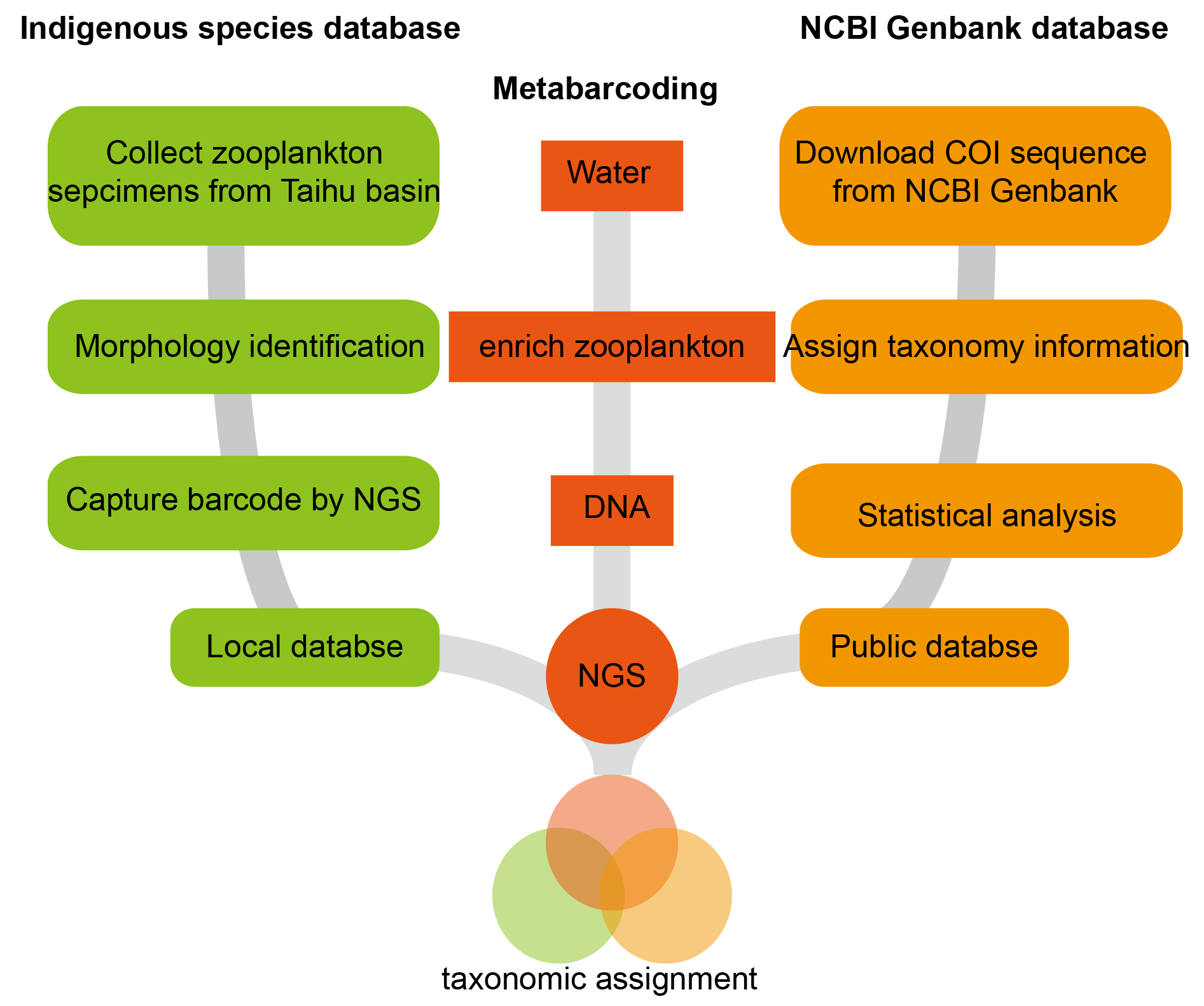

Supplement: S1 Fig — (TIF) [file pone.0185697.s002.tif]

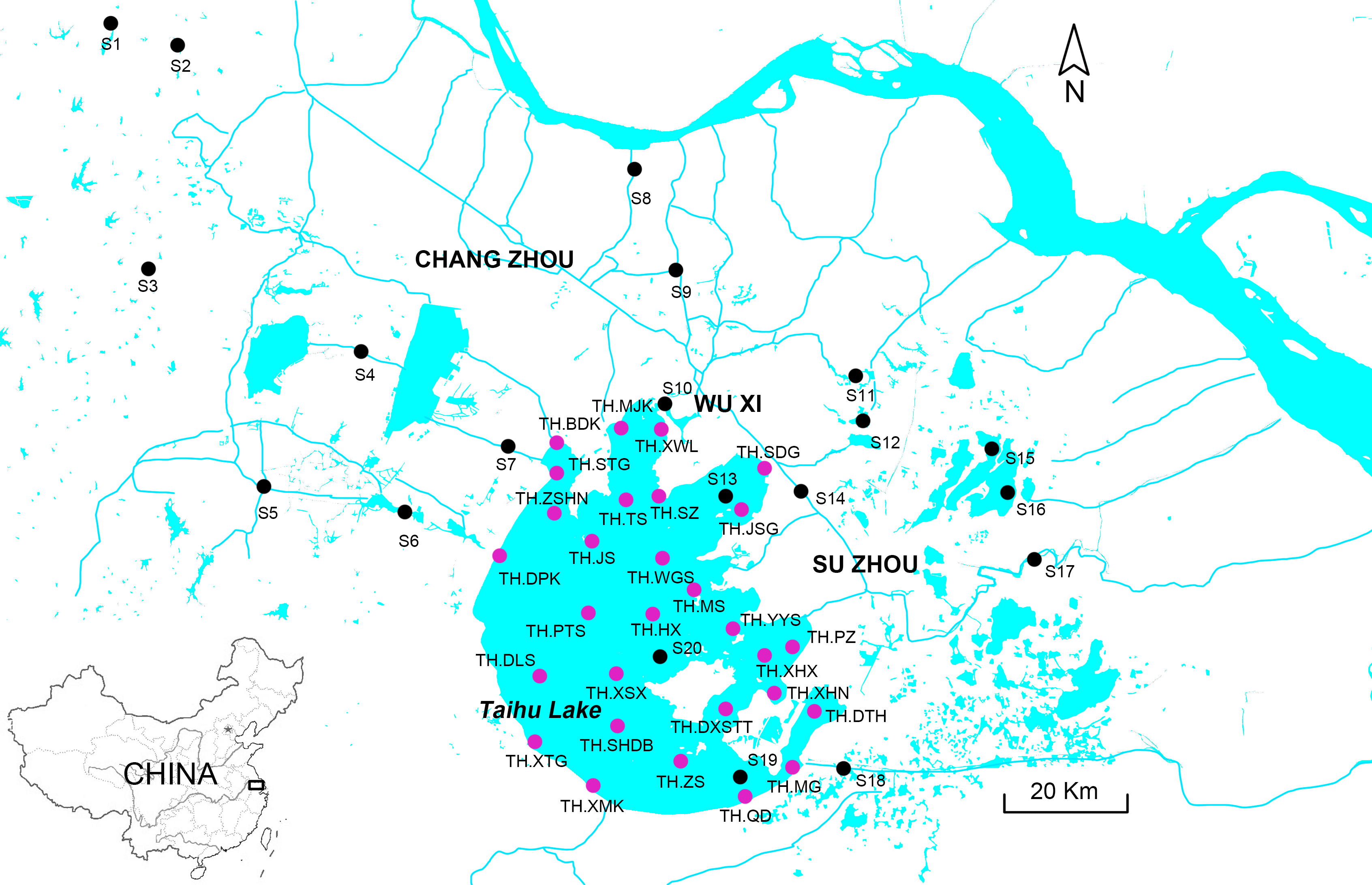

Supplement: S2 Fig — The sampling sites for indigenous barcode database were indicated by black dots. The sampling sites for zooplankton metabarcoding analysis were indicated by green dots. (TIF) [file pone.0185697.s003.tif]

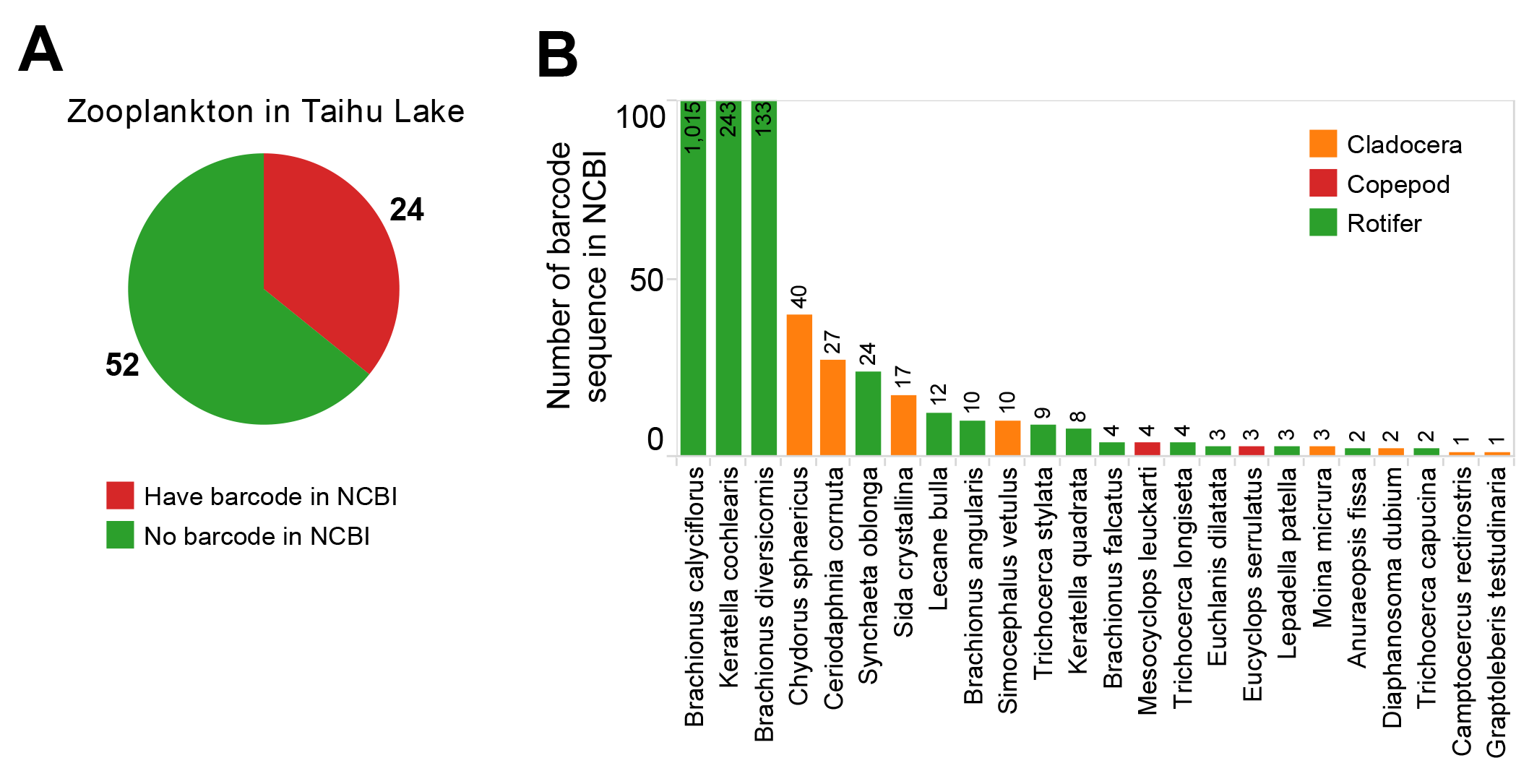

Supplement: S3 Fig — (A): the number of species. (B): the number of COI sequence. (TIF) [file pone.0185697.s004.tif]

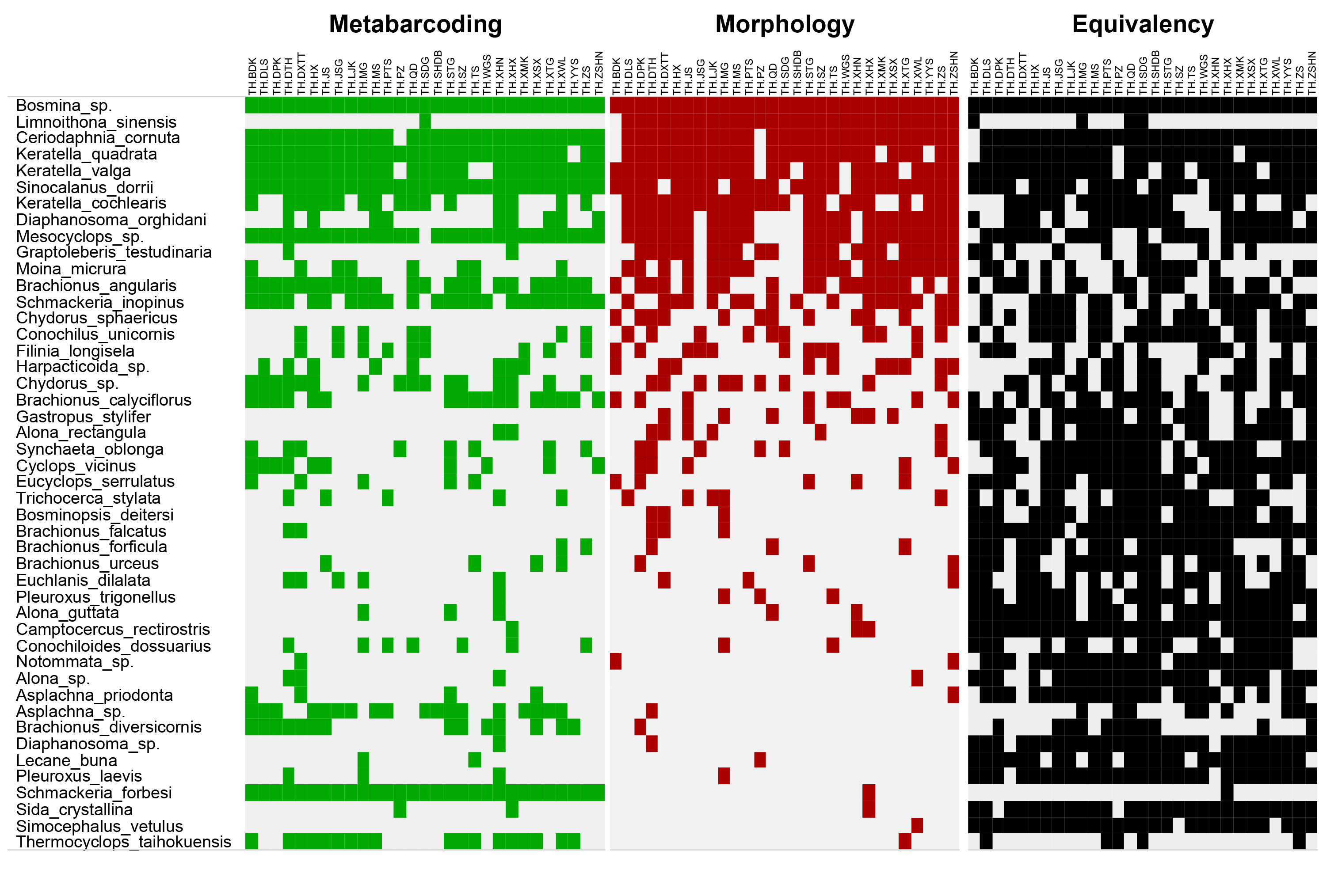

Supplement: S4 Fig — Green indicates presence for metabarcoding, red indicates presence for morphologic identification and white indicates not detected. For the equivalency, black indicates consistency of detection (presence or absence) of the same sample by both methods, white indicates inconsistency of specie detection. (TIF) [file pone.0185697.s005.tif]
